# Supplementary material for: Earlier Alzheimer’s disease onset is associated with tau pathology in brain hub regions and facilitated tau spreading
Source: Nat Commun. 2022 Aug 20;13:4899. doi: 10.1038/s41467-022-32592-7 (PMC9392750; doi:10.1038/s41467-022-32592-7)
Supplement: Supplementary file 1 — Supplementary Information [file 41467_2022_32592_MOESM1_ESM.pdf]

Supplementary table 1: Main analyses controlling for amyloid, ApoE4 or using robust regression

		ADNI		BioFINDER	
**Linear regression**	**Patient group**	**β**	**p-value**	**β**	**p-value**
Age vs. Tau hub ratio controlling for Global Aβ	preclinical	0.075	0.690	0.232	0.432
	Symptomatic	-0.229	0.034	-0.480	0.021
Age vs. Tau hub ratio controlling for ApoE4	preclinical	-0.019	0.898	-0.011	0.972
	Symptomatic	-0.214	0.049	-0.479	0.022
**Robust regression**	**Patient group**	**B/SE**	**p-value**	**B/SE**	**p-value**
Age vs. Tau hub ratio (robust regression)	preclinical	0.002/0.005	0.693	-0.0013/0.011	0.909
	Symptomatic	-0.008/0.379	0.045	-0.011/0.005	0.032

All analyses were controlled for age, sex and education. Analyses in Symptomatic AD patients were additionally controlled for diagnosis (i.e. MCI or dementia). β-values are standardized regression weights. All p-values are two-sided.

Supplementary table 2: Main analyses including hippocampal tau-PET

		ADNI		BioFINDER	
**Linear regression**	**Patient group**	**β**	**p-value**	**β**	**p-value**
Age vs. Tau hub ratio	preclinical	0.053	0.701	-0.135	0.650
	Symptomatic	-0.259	0.015	-0.452	0.027
Age of onset vs. tau hub ratio	Symptomatic	-0.269	0.019		
Age vs. tau epicenter hubness	preclinical	-0.227	0.107	0.089	0.767
	Symptomatic	0.344	<0.001	0.614	<0.001
Age vs. Tau accumulation rate	Preclinical	-0.121	0.380	0.210	0.436
	Symptomatic	-0.283	0.009	-0.836	<0.001
Tau hub ratio vs. tau accumulation rate	Preclinical	0.261	0.045	0.176	0.511
	Symptomatic	0.305	0.006	0.670	0.002
**ANCOVA**	**Patient group**	**F-value**	**p-value**	**F-value**	**p-value**
ApoE4 vs. tau hub ratio	AD (i.e. Aβ+)	4.609	0.045	4.248	0.042

All analyses were controlled for age, sex and education. Analyses in Symptomatic AD patients were additionally controlled for diagnosis (i.e. MCI or dementia). β-values are standardized regression weights. All p-values are two-sided.

Supplementary Figure 1:

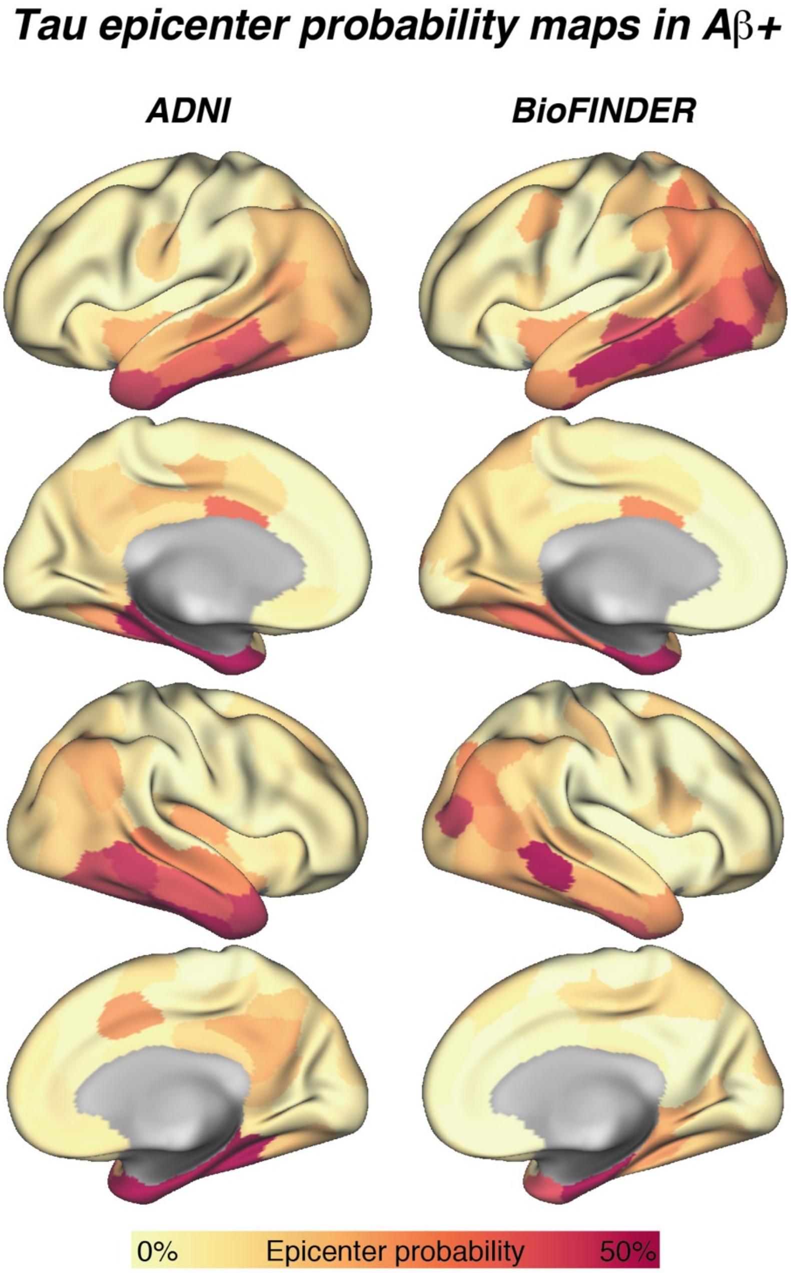

Supplementary Figure 1: Epicenter probability. Surface rendering of tau epicenter probability in Aβ+ subjects of the ADNI (left column) and BioFINDER (right column) sample. Source data are provided as a Source Data file.

Supplementary Figure 2:

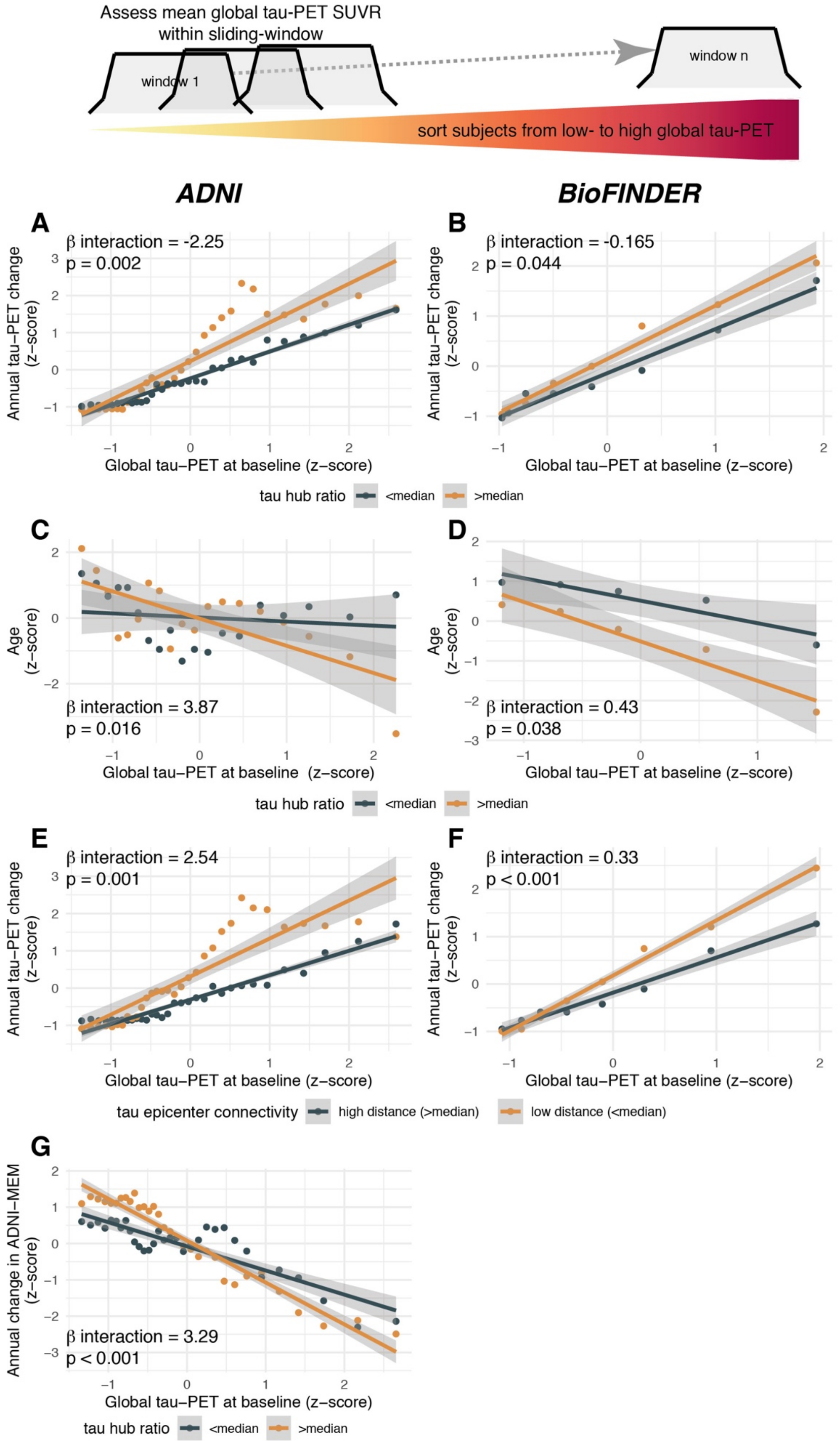

Supplementary Figure 2: Sliding window analyses. Sliding window analysis performed in Aβ+ subjects of the ADNI (n=147) and BioFINDER sample (n=41). Scatterplots of the interaction effect between baseline global tau-PET and the tau hub ratio on annual tau-PET change (A&B) and age (C&D). Scatterplots of the interaction effect

between baseline global tau-PET and tau epicenter connectivity on annual tau-PET change (E&F). Using longitudinal cognitive data in ADNI, we further tested the interaction between baseline global tau-PET and the tau hub ratio on annual changes in ADNI-MEM, i.e. a composite memory score. All statistical tests were performed using linear regression. β-values are standardized regression coefficients, two-sided p-values are based on linear regression. Linear model fits are indicated together with 95% confidence intervals. Source data are provided as a source data file.
