# Supplementary material for: Earlier Alzheimer’s disease onset is associated with tau pathology in brain hub regions and facilitated tau spreading
Source: Nat Commun. 2022 Aug 20;13:4899. doi: 10.1038/s41467-022-32592-7 (PMC9392750; doi:10.1038/s41467-022-32592-7)
Supplement: Supplementary file 3 — Description of Additional Supplementary Files [file 41467_2022_32592_MOESM3_ESM.pdf]

## Description of Additional Supplementary Files

**Supplementary Software 1:** A zip which includes all data and code that is required to recapitulate a simulated version of the analyses included in our manuscript.
