# Supplementary material for: Earlier Alzheimer’s disease onset is associated with tau pathology in brain hub regions and facilitated tau spreading
Source: Nat Commun. 2022 Aug 20;13:4899. doi: 10.1038/s41467-022-32592-7 (PMC9392750; doi:10.1038/s41467-022-32592-7)
Supplement: Supplementary file 4 — Supplementary Software 1 [file 41467_2022_32592_MOESM4_ESM.zip › Supplementary_Code/Example_Code_Output.html]

Example\_Code.utf8


```
library(corrplot)
library(lm.beta)
library(Hmisc)
library(qgraph)
library(plyr)
library(dplyr)
library(psych)
library(scales)
library(data.table)
library(ggplot2)
library(ggpubr)
library(ggsci)
library(readxl)
```

# data preparation

## > define input parameters

```
# number of subjects for simulation
n.subs = 100

# number of ROIs for simulation
n.ROIs = 200
```

## > Generate simulated data

```
set.seed(42)
# simulate baseline tau-PET
tau.pet = matrix(data = runif(n.subs*n.ROIs, 0,3), 
                 nrow = n.subs,
                 ncol = n.ROIs)
tau.pet.df = data.frame(tau.pet)
names(tau.pet.df) <- paste0("tau.pet.ROI", 1:n.ROIs)

# simulate tau-PET rate of change
tau.pet.roc = matrix(data = runif(n.subs*n.ROIs, 0,1), 
                 nrow = n.subs,
                 ncol = n.ROIs)
tau.pet.roc.df = data.frame(tau.pet.roc)
names(tau.pet.roc.df) <- paste0("tau.pet.ROI", 1:n.ROIs, ".ROC")

# simulate covariates
covariates.df = data.frame(age = runif(n.subs, 50,80),
                           education = runif(n.subs,8,17),
                           sex = sample(c("female", "male"), n.subs, replace = T),
                           ID = c(1:n.subs),
                           DX = sample(c("preclinical", "clinical"), n.subs, replace = T),
                           centiloid = runif(n.subs, 20,100),
                           cognition.ROC = runif(n.subs, -2,1),
                           epicenter.connectivity = rep(NA, n.subs))

# concatenate simulated dataset
full.data = cbind(covariates.df, tau.pet.df, tau.pet.roc.df)

# determine global tau-PET and tau-PET change rate
full.data$tau.pet.global.ROC = rowMeans(tau.pet.roc.df)
full.data$tau.pet.global = rowMeans(tau.pet)
```

## > generate simulated connectivity matrix and create scaled hub vector

```
set.seed(42)
# read in connectivity matrix
connectivity.distance = matrix(data = runif(n.ROIs*n.ROIs, 0,8), 
                               ncol = n.ROIs,
                               nrow = n.ROIs)


corrplot::corrplot(connectivity.distance, 
                   diag = FALSE, 
                   tl.pos = "n", 
                   tl.cex = 0.5, 
                   method = "color", 
                   is.corr = FALSE)
```

```
# create scaled hub vector
connectivity.degree.scaled = rescale(rank(rowMeans(connectivity.distance)), to = c(-1,1))
```

## > determine tau hub ratio

```
# select tau-PET data
tmp.tau.pet = as.matrix(full.data %>% select(paste0("tau.pet.ROI", 1:n.ROIs)))

# multiply subject-level tau-PET data with the scaled hub vector
tmp.tau.pet.weighted = sweep(tmp.tau.pet, MARGIN = 2, connectivity.degree.scaled, "*")

# create tau hub ratio adjusted for global tau levels
full.data$tau.hub.ratio = rowMeans(tmp.tau.pet.weighted)/rowMeans(tmp.tau.pet)
```

# determine epicenter connectivity

```
# run for loop across individuals
for (i in 1:nrow(full.data)){
  # select PET data
  current.pet = full.data[i, paste0("tau.pet.ROI", 1:n.ROIs)]
  
  # determine epicenters (i.e. 10% of brain regions with highest tau-PET)
  current.epicenters = which(ntile(current.pet, 10) == 10)
  
  # determine epicenter degree (i.e. hub connectivity)
  full.data$epicenter.connectivity[i] = 
    mean(rowMeans(connectivity.distance[-current.epicenters,current.epicenters]))
}
```

# > Main analysis

## > age vs. tau hub ratio - stratified by clinical status

```
# hub gradient
p_age_vs_tau_hub_ratio <- ggplot(data = full.data,
       aes( x = age,
            y = tau.hub.ratio,
            colour = DX)) + 
  geom_point(alpha = 0.5) + 
  geom_smooth(method = "lm") + scale_color_jama() + theme_minimal() + 
  facet_wrap(~DX, scales = "free_x")  + guides(colour = F)
print(p_age_vs_tau_hub_ratio)
```

```
## `geom_smooth()` using formula 'y ~ x'
```

```
# Association between age and tau hub ratio

# --- preclinical
tmp.lm <- lm.beta(lm(data = subset(full.data, DX == "preclinical"),
                     tau.hub.ratio ~ age + sex + education)); summary(tmp.lm)
```

```
## 
## Call:
## lm(formula = tau.hub.ratio ~ age + sex + education, data = subset(full.data, 
##     DX == "preclinical"))
## 
## Residuals:
##       Min        1Q    Median        3Q       Max 
## -0.045708 -0.015373  0.003292  0.011459  0.034798 
## 
## Coefficients:
##               Estimate Standardized Std. Error t value Pr(>|t|)
## (Intercept) -1.170e-02    0.000e+00  3.328e-02  -0.352    0.727
## age         -2.407e-05   -9.772e-03  3.845e-04  -0.063    0.950
## sexmale     -3.580e-03   -8.755e-02  6.266e-03  -0.571    0.571
## education    1.255e-03    1.608e-01  1.236e-03   1.016    0.316
## 
## Residual standard error: 0.02091 on 43 degrees of freedom
## Multiple R-squared:  0.03948,    Adjusted R-squared:  -0.02753 
## F-statistic: 0.5892 on 3 and 43 DF,  p-value: 0.6254
```

```
# --- clinical
tmp.lm <- lm.beta(lm(data = subset(full.data, DX == "clinical"),
                     tau.hub.ratio ~ age + sex + education)); summary(tmp.lm)
```

```
## 
## Call:
## lm(formula = tau.hub.ratio ~ age + sex + education, data = subset(full.data, 
##     DX == "clinical"))
## 
## Residuals:
##       Min        1Q    Median        3Q       Max 
## -0.044830 -0.016092  0.003113  0.017451  0.060194 
## 
## Coefficients:
##               Estimate Standardized Std. Error t value Pr(>|t|)
## (Intercept) -0.0392982    0.0000000  0.0315234  -1.247    0.218
## age          0.0002927    0.1009663  0.0004057   0.722    0.474
## sexmale     -0.0074999   -0.1500654  0.0070361  -1.066    0.292
## education    0.0012766    0.1445824  0.0012346   1.034    0.306
## 
## Residual standard error: 0.02527 on 49 degrees of freedom
## Multiple R-squared:  0.05423,    Adjusted R-squared:  -0.003672 
## F-statistic: 0.9366 on 3 and 49 DF,  p-value: 0.4302
```

## > age vs. epicenter connectivity - stratified by clinical status

```
# hub gradient
p_age_vs_epicenter_connectivity <- ggplot(data = full.data,
       aes( x = age,
            y = epicenter.connectivity,
            colour = DX)) + 
  geom_point(alpha = 0.5) + 
  geom_smooth(method = "lm") + scale_color_jama() + theme_minimal() + 
  facet_wrap(~DX, scales = "free_x")  + guides(colour = F)
print(p_age_vs_epicenter_connectivity)
```

```
## `geom_smooth()` using formula 'y ~ x'
```

```
# Association between age and tau hub ratio

# --- preclinical
tmp.lm <- lm.beta(lm(data = subset(full.data, DX == "preclinical"),
                     epicenter.connectivity ~ age + sex + education)); summary(tmp.lm)
```

```
## 
## Call:
## lm(formula = epicenter.connectivity ~ age + sex + education, 
##     data = subset(full.data, DX == "preclinical"))
## 
## Residuals:
##       Min        1Q    Median        3Q       Max 
## -0.066216 -0.019039  0.000903  0.021077  0.093798 
## 
## Coefficients:
##               Estimate Standardized Std. Error t value Pr(>|t|)    
## (Intercept)  3.9961227    0.0000000  0.0561726  71.140   <2e-16 ***
## age          0.0001954    0.0478822  0.0006491   0.301    0.765    
## sexmale     -0.0004282   -0.0063214  0.0105771  -0.040    0.968    
## education   -0.0001004   -0.0077645  0.0020869  -0.048    0.962    
## ---
## Signif. codes:  0 '***' 0.001 '**' 0.01 '*' 0.05 '.' 0.1 ' ' 1
## 
## Residual standard error: 0.03529 on 43 degrees of freedom
## Multiple R-squared:  0.002613,   Adjusted R-squared:  -0.06697 
## F-statistic: 0.03754 on 3 and 43 DF,  p-value: 0.9901
```

```
# --- clinical
tmp.lm <- lm.beta(lm(data = subset(full.data, DX == "clinical"),
                     epicenter.connectivity ~ age + sex + education)); summary(tmp.lm)
```

```
## 
## Call:
## lm(formula = epicenter.connectivity ~ age + sex + education, 
##     data = subset(full.data, DX == "clinical"))
## 
## Residuals:
##       Min        1Q    Median        3Q       Max 
## -0.057614 -0.017691 -0.001553  0.018107  0.095308 
## 
## Coefficients:
##               Estimate Standardized Std. Error t value Pr(>|t|)    
## (Intercept)  3.979e+00    0.000e+00  4.293e-02  92.698   <2e-16 ***
## age         -9.885e-05   -2.541e-02  5.524e-04  -0.179    0.859    
## sexmale      7.481e-03    1.115e-01  9.582e-03   0.781    0.439    
## education    1.534e-03    1.295e-01  1.681e-03   0.913    0.366    
## ---
## Signif. codes:  0 '***' 0.001 '**' 0.01 '*' 0.05 '.' 0.1 ' ' 1
## 
## Residual standard error: 0.03441 on 49 degrees of freedom
## Multiple R-squared:  0.02608,    Adjusted R-squared:  -0.03355 
## F-statistic: 0.4374 on 3 and 49 DF,  p-value: 0.7272
```

## > age vs. tau rate of change - stratified by clinical status

```
# hub gradient
p_age_vs_tau_ROC <- ggplot(data = full.data,
       aes( x = age,
            y = tau.pet.global.ROC,
            colour = DX)) + 
  geom_point(alpha = 0.5) + 
  geom_smooth(method = "lm") + scale_color_jama() + theme_minimal() + 
  facet_wrap(~DX, scales = "free_x")  + guides(colour = F)
print(p_age_vs_tau_ROC)
```

```
## `geom_smooth()` using formula 'y ~ x'
```

```
# Association between age and tau hub ratio

# --- preclinical
tmp.lm <- lm.beta(lm(data = subset(full.data, DX == "preclinical"),
                     tau.pet.global.ROC ~ age + sex + education)); summary(tmp.lm)
```

```
## 
## Call:
## lm(formula = tau.pet.global.ROC ~ age + sex + education, data = subset(full.data, 
##     DX == "preclinical"))
## 
## Residuals:
##      Min       1Q   Median       3Q      Max 
## -0.05428 -0.00962 -0.00120  0.01140  0.05294 
## 
## Coefficients:
##               Estimate Standardized Std. Error t value Pr(>|t|)    
## (Intercept)  0.4645243    0.0000000  0.0349852  13.278   <2e-16 ***
## age          0.0005964    0.2279025  0.0004043   1.475    0.147    
## sexmale     -0.0019456   -0.0447911  0.0065876  -0.295    0.769    
## education   -0.0002285   -0.0275603  0.0012997  -0.176    0.861    
## ---
## Signif. codes:  0 '***' 0.001 '**' 0.01 '*' 0.05 '.' 0.1 ' ' 1
## 
## Residual standard error: 0.02198 on 43 degrees of freedom
## Multiple R-squared:  0.05898,    Adjusted R-squared:  -0.006668 
## F-statistic: 0.8984 on 3 and 43 DF,  p-value: 0.4498
```

```
# --- clinical
tmp.lm <- lm.beta(lm(data = subset(full.data, DX == "clinical"),
                     tau.pet.global.ROC ~ age + sex + education)); summary(tmp.lm)
```

```
## 
## Call:
## lm(formula = tau.pet.global.ROC ~ age + sex + education, data = subset(full.data, 
##     DX == "clinical"))
## 
## Residuals:
##       Min        1Q    Median        3Q       Max 
## -0.037747 -0.012170  0.003107  0.011187  0.037379 
## 
## Coefficients:
##               Estimate Standardized Std. Error t value Pr(>|t|)    
## (Intercept)  5.162e-01    0.000e+00  2.371e-02  21.777   <2e-16 ***
## age         -5.896e-05   -2.762e-02  3.051e-04  -0.193    0.848    
## sexmale     -2.124e-03   -5.771e-02  5.291e-03  -0.401    0.690    
## education   -6.787e-04   -1.044e-01  9.284e-04  -0.731    0.468    
## ---
## Signif. codes:  0 '***' 0.001 '**' 0.01 '*' 0.05 '.' 0.1 ' ' 1
## 
## Residual standard error: 0.019 on 49 degrees of freedom
## Multiple R-squared:  0.01388,    Adjusted R-squared:  -0.04649 
## F-statistic: 0.2299 on 3 and 49 DF,  p-value: 0.8751
```

## > tau hub ratio vs. tau-PET rate of change - stratified by clinical status

```
# hub gradient
p_tau_hub_ratio_vs_tau_ROC <- ggplot(data = full.data,
       aes( x = tau.hub.ratio,
            y = tau.pet.global.ROC,
            colour = DX)) + 
  geom_point(alpha = 0.5) + 
  geom_smooth(method = "lm") + scale_color_jama() + theme_minimal() + 
  facet_wrap(~DX, scales = "free_x")  + guides(colour = F)
print(p_tau_hub_ratio_vs_tau_ROC)
```

```
## `geom_smooth()` using formula 'y ~ x'
```

```
# Association between age and tau hub ratio

# --- preclinical
tmp.lm <- lm.beta(lm(data = subset(full.data, DX == "preclinical"),
                     tau.pet.global.ROC ~ tau.hub.ratio + sex + education)); summary(tmp.lm)
```

```
## 
## Call:
## lm(formula = tau.pet.global.ROC ~ tau.hub.ratio + sex + education, 
##     data = subset(full.data, DX == "preclinical"))
## 
## Residuals:
##       Min        1Q    Median        3Q       Max 
## -0.054947 -0.013300  0.000971  0.010070  0.059613 
## 
## Coefficients:
##                 Estimate Standardized Std. Error t value Pr(>|t|)    
## (Intercept)    0.5096966    0.0000000  0.0170804  29.841   <2e-16 ***
## tau.hub.ratio -0.0231603   -0.0218044  0.1642902  -0.141    0.889    
## sexmale       -0.0032397   -0.0745822  0.0067230  -0.482    0.632    
## education     -0.0007366   -0.0888426  0.0012956  -0.569    0.573    
## ---
## Signif. codes:  0 '***' 0.001 '**' 0.01 '*' 0.05 '.' 0.1 ' ' 1
## 
## Residual standard error: 0.02253 on 43 degrees of freedom
## Multiple R-squared:  0.01182,    Adjusted R-squared:  -0.05712 
## F-statistic: 0.1715 on 3 and 43 DF,  p-value: 0.9151
```

```
# --- clinical
tmp.lm <- lm.beta(lm(data = subset(full.data, DX == "clinical"),
                     tau.pet.global.ROC ~ tau.hub.ratio + sex + education)); summary(tmp.lm)
```

```
## 
## Call:
## lm(formula = tau.pet.global.ROC ~ tau.hub.ratio + sex + education, 
##     data = subset(full.data, DX == "clinical"))
## 
## Residuals:
##       Min        1Q    Median        3Q       Max 
## -0.037800 -0.012822  0.003223  0.011696  0.036568 
## 
## Coefficients:
##                 Estimate Standardized Std. Error t value Pr(>|t|)    
## (Intercept)    0.5128341    0.0000000  0.0127063  40.361   <2e-16 ***
## tau.hub.ratio  0.0250685    0.0340403  0.1068406   0.235    0.815    
## sexmale       -0.0020705   -0.0562565  0.0053053  -0.390    0.698    
## education     -0.0007088   -0.1090037  0.0009380  -0.756    0.453    
## ---
## Signif. codes:  0 '***' 0.001 '**' 0.01 '*' 0.05 '.' 0.1 ' ' 1
## 
## Residual standard error: 0.019 on 49 degrees of freedom
## Multiple R-squared:  0.01424,    Adjusted R-squared:  -0.04612 
## F-statistic: 0.2359 on 3 and 49 DF,  p-value: 0.8709
```

## > Mediation age > tau hub ratio > tau-PET rate of change

```
set.seed(42)
# age on tau hub gradient
tmp.lm.effect.on.mediator <- lm(data = subset(full.data, DX == "clinical"),
                     scale(tau.hub.ratio) ~ scale(age) + sex + education); summary(tmp.lm.effect.on.mediator)
```

```
## 
## Call:
## lm(formula = scale(tau.hub.ratio) ~ scale(age) + sex + education, 
##     data = subset(full.data, DX == "clinical"))
## 
## Residuals:
##     Min      1Q  Median      3Q     Max 
## -1.7773 -0.6380  0.1234  0.6918  2.3864 
## 
## Coefficients:
##             Estimate Std. Error t value Pr(>|t|)
## (Intercept) -0.49158    0.66056  -0.744    0.460
## scale(age)   0.10097    0.13993   0.722    0.474
## sexmale     -0.29734    0.27895  -1.066    0.292
## education    0.05061    0.04895   1.034    0.306
## 
## Residual standard error: 1.002 on 49 degrees of freedom
## Multiple R-squared:  0.05423,    Adjusted R-squared:  -0.003672 
## F-statistic: 0.9366 on 3 and 49 DF,  p-value: 0.4302
```

```
# age on accumulation rate while adjusting for hub gradient
tmp.lm.indirect.effect <- lm(data = subset(full.data, DX == "clinical"),
                     scale(tau.pet.global.ROC) ~ scale(age) + scale(tau.hub.ratio) + sex + education); summary(tmp.lm.indirect.effect)
```

```
## 
## Call:
## lm(formula = scale(tau.pet.global.ROC) ~ scale(age) + scale(tau.hub.ratio) + 
##     sex + education, data = subset(full.data, DX == "clinical"))
## 
## Residuals:
##     Min      1Q  Median      3Q     Max 
## -2.0349 -0.6896  0.1684  0.6183  1.9583 
## 
## Coefficients:
##                      Estimate Std. Error t value Pr(>|t|)
## (Intercept)           0.53462    0.68488   0.781    0.439
## scale(age)           -0.03138    0.14503  -0.216    0.830
## scale(tau.hub.ratio)  0.03731    0.14729   0.253    0.801
## sexmale              -0.10325    0.29091  -0.355    0.724
## education            -0.03843    0.05101  -0.753    0.455
## 
## Residual standard error: 1.033 on 48 degrees of freedom
## Multiple R-squared:  0.0152, Adjusted R-squared:  -0.06687 
## F-statistic: 0.1852 on 4 and 48 DF,  p-value: 0.945
```

```
# bootstrapped mediation
# Estimation via quasi-Bayesian approximation
mediation.model <- mediation::mediate(model.m = tmp.lm.effect.on.mediator, 
                           model.y =  tmp.lm.indirect.effect, 
                           ims=1000, 
                           treat="scale(age)",
                           mediator="scale(tau.hub.ratio)")
```

```
## Registered S3 methods overwritten by 'lme4':
##   method                          from
##   cooks.distance.influence.merMod car 
##   influence.merMod                car 
##   dfbeta.influence.merMod         car 
##   dfbetas.influence.merMod        car
```

```
summary(mediation.model)
```

```
## 
## Causal Mediation Analysis 
## 
## Quasi-Bayesian Confidence Intervals
## 
##                Estimate 95% CI Lower 95% CI Upper p-value
## ACME            0.00403     -0.04683         0.07    0.91
## ADE            -0.03167     -0.31049         0.26    0.81
## Total Effect   -0.02764     -0.31526         0.27    0.84
## Prop. Mediated  0.00142     -1.58671         1.88    0.98
## 
## Sample Size Used: 53 
## 
## 
## Simulations: 1000
```

```
plot(mediation.model)
```

## > global tau-PET vs. cognitive rate of change

```
# hub gradient
p_tau_pet_global_vs_cognition_ROC <- ggplot(data = full.data,
       aes( x = tau.pet.global,
            y = cognition.ROC)) + 
  geom_point(alpha = 0.5) + 
  geom_smooth(method = "lm") + scale_color_jama() + theme_minimal() + guides(colour = F)
print(p_tau_pet_global_vs_cognition_ROC)
```

```
## `geom_smooth()` using formula 'y ~ x'
```

```
# Association between tau-PET and cognitive rate of change
tmp.lm <- lm.beta(lm(data = subset(full.data, DX == "preclinical"),
                     cognition.ROC ~ tau.pet.global + age + sex + education)); summary(tmp.lm)
```

```
## 
## Call:
## lm(formula = cognition.ROC ~ tau.pet.global + age + sex + education, 
##     data = subset(full.data, DX == "preclinical"))
## 
## Residuals:
##      Min       1Q   Median       3Q      Max 
## -1.48081 -0.62227 -0.09675  0.59052  1.89365 
## 
## Coefficients:
##                 Estimate Standardized Std. Error t value Pr(>|t|)  
## (Intercept)    -4.891247     0.000000   2.965556  -1.649   0.1065  
## tau.pet.global  3.304727     0.254394   1.960084   1.686   0.0992 .
## age            -0.007023    -0.069266   0.015751  -0.446   0.6580  
## sexmale        -0.379770    -0.225634   0.251145  -1.512   0.1380  
## education      -0.007158    -0.022279   0.049290  -0.145   0.8852  
## ---
## Signif. codes:  0 '***' 0.001 '**' 0.01 '*' 0.05 '.' 0.1 ' ' 1
## 
## Residual standard error: 0.8304 on 42 degrees of freedom
## Multiple R-squared:  0.1263, Adjusted R-squared:  0.04311 
## F-statistic: 1.518 on 4 and 42 DF,  p-value: 0.2143
```
