# Supplementary material for: Earlier Alzheimer’s disease onset is associated with tau pathology in brain hub regions and facilitated tau spreading
Source: Nat Commun. 2022 Aug 20;13:4899. doi: 10.1038/s41467-022-32592-7 (PMC9392750; doi:10.1038/s41467-022-32592-7)
Supplement: Supplementary file 5 — Reporting Summary [file 41467_2022_32592_MOESM5_ESM.pdf]

nature portfolio

Corresponding author(s): Dr. Nicolai Franzmeier

Last updated by author(s): July 4th, 2022

Reporting Summary

Nature Portfolio wishes to improve the reproducibility of the work that we publish. This form provides structure for consistency and transparency in reporting. For further information on Nature Portfolio policies, see our Editorial Policies and the Editorial Policy Checklist.

Statistics

For all statistical analyses, confirm that the following items are present in the figure legend, table legend, main text, or Methods section.

n/a	Confirmed	
☐	☒	The exact sample size (n) for each experimental group/condition, given as a discrete number and unit of measurement
☐	☒	A statement on whether measurements were taken from distinct samples or whether the same sample was measured repeatedly
☐	☒	The statistical test(s) used AND whether they are one- or two-sided<br>*Only common tests should be described solely by name; describe more complex techniques in the Methods section.*
☐	☒	A description of all covariates tested
☐	☒	A description of any assumptions or corrections, such as tests of normality and adjustment for multiple comparisons
☐	☒	A full description of the statistical parameters including central tendency (e.g. means) or other basic estimates (e.g. regression coefficient) AND variation (e.g. standard deviation) or associated estimates of uncertainty (e.g. confidence intervals)
☐	☒	For null hypothesis testing, the test statistic (e.g. F, t, r) with confidence intervals, effect sizes, degrees of freedom and P value noted<br>*Give P values as exact values whenever suitable.*
☒	☐	For Bayesian analysis, information on the choice of priors and Markov chain Monte Carlo settings
☒	☐	For hierarchical and complex designs, identification of the appropriate level for tests and full reporting of outcomes
☐	☒	Estimates of effect sizes (e.g. Cohen's d, Pearson's r), indicating how they were calculated

Our web collection on statistics for biologists contains articles on many of the points above.

Software and code

Policy information about availability of computer code

Data collection	no software was used
Data analysis	All analyses were computed using R statistical software 4.0.4 and the Mediation package Version 4.5.0. Example code and simulated data have been uploaded as a supplementary file

For manuscripts utilizing custom algorithms or software that are central to the research but not yet described in published literature, software must be made available to editors and reviewers. We strongly encourage code deposition in a community repository (e.g. GitHub). See the Nature Portfolio guidelines for submitting code & software for further information.

Data

Policy information about availability of data

All manuscripts must include a data availability statement. This statement should provide the following information, where applicable:

- Accession codes, unique identifiers, or web links for publicly available datasets
- A description of any restrictions on data availability
- For clinical datasets or third party data, please ensure that the statement adheres to our policy

All data used in this manuscript are publicly available from the ADNI database (adni.loni.usc.edu) upon registration and compliance with the data use agreement. BioFINDER data are available from the principal investigator (OH), anonymized data will be shared by request from a qualified academic investigator for the sole purpose of replicating procedures and results presented in the article and as long as data transfer is in agreement with EU legislation on the general data protection regulation and decisions by the Ethical Review Board of Sweden and Region Skåne, which should be regulated in a material transfer agreement. The data that support the findings of this study are available on reasonable request from the corresponding author. Source data are provided with this paper.

Field-specific reporting

Please select the one below that is the best fit for your research. If you are not sure, read the appropriate sections before making your selection.

- [x] Life sciences
- [ ] Behavioural & social sciences
- [ ] Ecological, evolutionary & environmental sciences

For a reference copy of the document with all sections, see nature.com/documents/nr-reporting-summary-flat.pdf

Life sciences study design

All studies must disclose on these points even when the disclosure is negative.

Sample size	ADNI, n = 240, BioFINDER, n = 57; sample sizes were determined based on maximum available data that met the inclusion criteria. No specific statistical methods or sample size estimations were used to define sample size
Data exclusions	No data were excluded from the analyses
Replication	Findings were validated across two independent datasets
Randomization	This was an observational study without any intervention, so no randomization was performed
Blinding	This was a study that retrospectively analyzed available observational clinical and neuroimaging data without any intervention, so no blinding was performed

Reporting for specific materials, systems and methods

We require information from authors about some types of materials, experimental systems and methods used in many studies. Here, indicate whether each material, system or method listed is relevant to your study. If you are not sure if a list item applies to your research, read the appropriate section before selecting a response.

Materials & experimental systems

n/a	Involved in the study	
x		Antibodies
x		Eukaryotic cell lines
x		Palaeontology and archaeology
x		Animals and other organisms
	x	Human research participants
	x	Clinical data
x		Dual use research of concern

Methods

n/a	Involved in the study	
x		ChIP-seq
x		Flow cytometry
	x	MRI-based neuroimaging

Human research participants

Policy information about studies involving human research participants

Population characteristics	Participants - ADNI:<br>We included 240 participants of the Alzheimer's Disease Neuroimaging Initiative (ADNI) database. Inclusion criteria were availability of longitudinal 18F-Flortaucipir tau-PET as well as baseline 18F-Florbetapir or 18F-Florbetaben amyloid-PET, T1 MPRAGE structural MRI and demographic data. All baseline imaging had to be collected within 12 months. A-status was assessed based on whole-cerebellum normalized global amyloid-PET SUVR, using pre-established protocols and cut-points (global AV45 SUVR>1.11; global FBB SUVR>1.08).70 Global amyloid-PET SUVRs were further transformed to the Centiloid scale to allow pooling across tracers.71 Clinical status was assessed by ADNI, categorizing subjects as cognitively normal (CN, MMSE>24, CDR=0, non-depressed), mild cognitively impaired (MCI, MMSE>24, CDR=0.5, objective memory-impairment on education adjusted Wechsler Memory Scale II, preserved activities of daily living), or demented (MMSE 20-26, CDR>0.5, NINCDS/ADRDA criteria for probable AD). Amyloid-positive (n=149) individuals were grouped as preclinical AD (i.e. cognitively normal, n=60) or AD clinical syndrome (i.e. MCI [n=57] & Dementia[n=32]). Ethical approval was obtained by the ADNI investigators, all participants provided written informed consent. All study relevant covariates are described in table 1.<br><br>Participants - BioFINDER:<br>As a replication sample, we included 57 BioFINDER participants with available A-status, structural MRI and longitudinal 18F-Flortaucipir tau-PET. Baseline A-status was determined using 18F-Flutemetamol-PET as described previously 57, applying a pons-normalized global SUVR cut-off>0.575 72. The Alzheimer's continuum was covered by 16 CN A+, 7 MCI A+ and 18 AD dementia subjects vs. 16 CN A- subjects as controls. BioFINDER inclusion and exclusion criteria as well as diagnostic criteria have been described previously 73. As for ADNI, amyloid-positive individuals were grouped as preclinical AD (i.e. cognitively normal) or AD clinical syndrome (i.e. MCI & Dementia). All participants provided written informed consent prior to study inclusion. Ethical approval was provided by the ethics committee at Lund University, Sweden. Imaging procedures were approved by the Radiation protection committee at Skåne University Hospital and by the Swedish Medical Products Agency. All study relevant covariates are described in table 1.
Recruitment	ADNI patients are recruited within the north american ADNI study from specialized centers that participate in ADNI. Details on ADNI recruitment, inclusion criteria and study design can be found on the ADNI website (https://adni.loni.usc.edu/methods/documents/). BioFINDER participants were recruited at Lund University in Sweden from the local memory clinic. Details on BioFINDER recruitment and settings can be found online (https://biofinder.se)
Ethics oversight	For BioFINDER, ethical approval was provided by the ethics committee at Lund University, Sweden. Imaging procedures were approved by the Radiation protection committee at Skåne University Hospital and by the Swedish Medical Products Agency. For ADNI, ethical approval was obtained by the ADNI investigators at each participating site, all participants provided written informed consent.

Note that full information on the approval of the study protocol must also be provided in the manuscript.

Clinical data

Policy information about clinical studies

All manuscripts should comply with the ICMJE guidelines for publication of clinical research and a completed CONSORT checklist must be included with all submissions.

Clinical trial registration	ADNI, ClinicalTrials.gov Identifier: NCT02854033; BioFINDER, ClinicalTrials.gov Identifier: NCT01208675
Study protocol	The BioFINDER study protocol can be obtained from the authors upon request. The ADNI study protocol can be found online at https://adni.loni.usc.edu/methods/documents/
Data collection	All imaging and clinical data were collected at participating ADNI sites (between 2015-2020) and at the Skane University Hospital (between 2014-2018).
Outcomes	This was an observational study that retrospectively analyzed available longitudinal datasets, hence no specific outcomes were defined a priori

Magnetic resonance imaging

Experimental design

Design type	structural MRI, resting-state fMRI
Design specifications	no specific task was used
Behavioral performance measures	No behavioral measures were obtained during MRI scanning

Acquisition

Imaging type(s): T1-weighted structural MRI, resting-state functional MRI (Echo-Planar Imaging)

Field strength: 3

Sequence & imaging parameters: T1, Echo Planar Imaging

Area of acquisition: brain

Diffusion MRI: ☐ Used ☒ Not used

Preprocessing

Preprocessing software: ANTs, FSL

Normalization: ANTs

Normalization template: MNI

Noise and artifact removal: fMRI: To denoise the EPI images, we regressed out nuisance covariates (i.e. average white matter and cerebrospinal fluid signal and motion parameters estimated during motion correction), removed the linear trend and applied band-pass filtering with a 0.01-0.08Hz frequency band in EPI native space. To further minimize the impact of motion which may compromise FC assessment, we performed motion scrubbing, where we censored volumes that showed a frame-wise displacement of >1mm, as well as one prior and two subsequent volumes. In line with our previous work, only subjects for whom less than 30% of volumes had to be censored were included in the current study.

Volume censoring: To further minimize the impact of motion which may compromise FC assessment, we performed motion scrubbing, where we censored volumes that showed a frame-wise displacement of >1mm, as well as one prior and two subsequent volumes. In line with our previous work, only subjects for whom less than 30% of volumes had to be censored were included in the current study.

Statistical modeling & inference

Model type and settings: No voxel-wise or cluster level analyses using fMRI data were conducted, fMRI data was not used as an outcome or dependent variable in any of the statistical analyses

Effect(s) tested: see above

Specify type of analysis: ☐ Whole brain ☒ ROI-based ☐ Both

Anatomical location(s): Schaefer Atlas

Statistic type for inference (See Eklund et al. 2016): No voxel level or mass univariate analyses were conducted using fMRI or MRI data, hence no cluster-wise statistical inference was applied

Correction: No voxel level or mass univariate analyses were conducted, see above

Models & analysis

n/a	Involved in the study
☐	☒ Functional and/or effective connectivity
☒	☐ Graph analysis
☒	☐ Multivariate modeling or predictive analysis

Functional and/or effective connectivity: Fisher-z transformed Pearson Correlation, which was thresholded at 30% and transformed to connectivity-based distance
